# FGF21 and the Physiological Regulation of Macronutrient Preference

**DOI:** 10.1210/endocr/bqaa019

**Published:** 2020-02-12

**Authors:** Cristal M Hill, Emily Qualls-Creekmore, Hans-Rudolf Berthoud, Paul Soto, Sangho Yu, David H McDougal, Heike Münzberg, Christopher D Morrison

**Affiliations:** Pennington Biomedical Research Center, Baton Rouge, LA

**Keywords:** protein, food choice, macronutrient, feeding behavior, nutrient sensing, homeostasis

## Abstract

The ability to respond to variations in nutritional status depends on regulatory systems that monitor nutrient intake and adaptively alter metabolism and feeding behavior during nutrient restriction. There is ample evidence that the restriction of water, sodium, or energy intake triggers adaptive responses that conserve existing nutrient stores and promote the ingestion of the missing nutrient, and that these homeostatic responses are mediated, at least in part, by nutritionally regulated hormones acting within the brain. This review highlights recent research that suggests that the metabolic hormone fibroblast growth factor 21 (FGF21) acts on the brain to homeostatically alter macronutrient preference. Circulating FGF21 levels are robustly increased by diets that are high in carbohydrate but low in protein, and exogenous FGF21 treatment reduces the consumption of sweet foods and alcohol while alternatively increasing the consumption of protein. In addition, while control mice adaptively shift macronutrient preference and increase protein intake in response to dietary protein restriction, mice that lack either FGF21 or FGF21 signaling in the brain fail to exhibit this homeostatic response. FGF21 therefore mediates a unique physiological niche, coordinating adaptive shifts in macronutrient preference that serve to maintain protein intake in the face of dietary protein restriction.

In recent decades there has been tremendous progress in defining the endocrine and neural mechanisms that control food intake in response to variations in nutritional status. The discovery of hormones such as leptin, which act in the brain to control feeding and energy balance, has triggered a broad effort that collectively has identified of a number of centrally acting endocrine signals as well as a variety of neural populations and circuits that respond to circulating nutritional cues and alter food intake when stimulated or inhibited (1–9).

Despite this progress, it must be acknowledged that much of this work has focused on food intake in a relatively univariate manner. Mice, rats, or other model organisms are offered a single diet, and thus their only option is to vary total food intake. Changes in total food intake (energy intake) are undeniably important and of significant relevance to body weight homeostasis and obesity. However, experiments that provide only one food option omit a key aspect of feeding that is highly relevant to humans—choice. Free-feeding animals, including humans, are rarely exposed to only a single diet or food source, but instead must navigate a complex nutritional landscape in which food sources differ in energy density, nutrient composition, palatability, availability, procurement cost, etc. Navigating such an environment requires that animals make choices that maximize nutrient intake, minimize procurement cost and risk, and adaptively respond to changes in either physiological state or environmental conditions. These choices require prioritization and compromise, as few foods provide an ideal nutrient composition. Considering the importance and complexity of choice in the context of feeding, this review focuses in particular on macronutrient choice and the potential role of the metabolic hormone FGF21 as a physiological signal that guides macronutrient choice in order to balance protein and carbohydrate.

## Vigorous Physiological Defense Against Nutrient Restriction: Fluid, Sodium, and Energy

Thriving within a complex and demanding environment requires that animals identify and consume foods that meet their nutritional needs. It is therefore almost self-evident that physiological systems must exist that identify nutritional deficits and trigger metabolic and behavioral adaptations that mitigate the deficit. However, “nutrition” is an incredibly complex parameter made up of a host of endpoints, and it would be a daunting task if every single nutrient were constantly monitored and defended. Here we briefly review the evidence that animals prioritize and defend a select set of primary nutritional variables.

Adequate fluid intake is essential for survival, consistent with the oft-mentioned fact that a human can last days or even weeks without food but will perish in only a few days without water. Fluid restriction generates a robust physiological response, which includes metabolic adaptations that promote fluid conservation and behavioral adaptations that promote fluid consumption. Substantial progress has been made in defining the neurobiological mechanisms mediating this thirst response, with neurons within subfornical organ (SFO), median preoptic nuclei (MnPO), and organum vasculosum of the lamina terminalis (OVLT) playing a critical role in sensing fluid restriction, organizing subsequent drinking behavior, altering vasopressin secretion through downstream projections, and finally detecting repletion following fluid ingestion (10–15). Body fluid regulation is also closely tied to salt/sodium balance, with neurons in the lamina terminalis, hypothalamus, and brainstem playing a key role in sodium appetite (12, 16, 17). Sodium depletion causes a robust and highly specific motivation to consume sodium, such that salt solutions that are negative/aversive in the replete state become preferred/rewarding in sodium-depleted animals (18–21). Sodium appetite has become the prototypical example of an intrinsic appetite that is dynamically dependent on need state, with sodium depletion increasing the rewarding value of sodium via an innate, taste-guided phenomenon that manifests without the need for learning or prior experience. Fluid balance is absolutely critical for survival, and as such the defense of fluid balance/sodium ranks as one of the clearest and strongest examples of a neural system mediating the behavioral defense of a nutrient-specific appetite.

It is well accepted that animals also sense and respond to the restriction of energy. Such a response provides a clear adaptive advantage considering that food availability can be intermittent and unpredictable, energy density can vary substantially between food sources, and the expenditure of energy due to physical activity or thermoregulation can vary across days or seasons. It therefore seems logical that a regulatory system exists that detects the onset of negative energy balance and adaptively controls metabolism and feeding behavior to meet ongoing energy needs. Past decades have seen an explosion in our understanding of the endocrine and neural mechanisms mediating the adaptive response to energy restriction. The study of central leptin action has played a pivotal and organizing role in this effort, but leptin is just one of many hormones that contribute to the defensive response to weight loss (3–5, 9, 22–24). Similarly, a large number of interacting neural circuits and brain areas have been linked to the regulation of feeding, including hypothalamic areas associated with feeding and body weight homeostasis; brainstem areas associated with the interoceptive detection of gut-derived signals and the resultant effects on meal size and satiation; and finally brain areas associated with complex behaviors including reward, motivation, and memory (1, 6–8, 25–28). Thus, it is now well accepted that animals sense and adaptively respond to the restriction of energy, and that this adaptive response is mediated principally by endocrine hormones that act directly in the brain as signals of energy status.

## Macronutrients: Do We Defend Against the Restriction of Fat, Carbohydrate or Protein?

Food intake is generally captured as a measurement of mass (grams) consumed, with this value at times being normalized to energy intake (calories/joules). Since most studies in rodent models provide only a single diet, there is also little value in viewing intake in any other way because nutrient composition is locked into a fixed proportion. It is only when multiple diets are offered which differ in their macronutrient composition that an assessment of macronutrient intake becomes relevant. Even in this situation, the study of macronutrient intake is problematic because as one nutrient (say protein) is reduced, another nutrient (say carbohydrate) must be increased in order to maintain equal energy density. Thus, it can be difficult to discern whether animals are choosing for one nutrient or against another. Any manipulation of dietary fat produces the added complication of altering energy density, and thus the researcher must decide whether nonnutritive filler must be included to control energy density. Together these issues make the experimental investigation of diet a complex design game in which it is nearly impossible to control all variables.

To combat these issues, scientists have sought approaches to test macronutrient intake in isolation. A classic example is the 3-food choice test, in which animals are offered, either together or in isolation, a source of pure protein (casein), pure fat (lard or oil), and/or pure carbohydrate (maltodextrin or sucrose), in either solid or liquid forms. Such a design provides substantial power by offering each nutrient in a pure form, independent of the other macronutrients. However, a concern is that the nutrients are offered in a form that is not representative of how they are experienced in a more natural setting (ie, as a mixed diet). In pure form, the sensory properties and palatability of nutrients are particularly exaggerated. Thus it can be problematic if studies are comparing the consumption of a low palatable source (solid casein) and a high palatable source (liquid sucrose).

One approach to combat this issue is to offer a series of diets covering a spectrum of macronutrient compositions, an approach best summarized by the nutritional geometric framework approach (29–32). The multidimensional nature of this geometric modeling allows one to assess shifts in macronutrient consumption (or any other dietary component) while also capturing the more complex interactions between nutrients. However, the effectiveness of this approach is maximized when a large number of dietary combinations are compared, which of course requires a large number of animal groups and diets. While nutritional geometry is an incredibly powerful tool, its adoption has been slowed by the necessity of multiple dietary groups and a more complicated analysis.

### The defense against carbohydrate and fat restriction appears to be weak

The maintenance of blood glucose concentrations is clearly under intense physiological regulation, and as such one might speculate that animals defend against carbohydrate restriction. Yet the restriction of dietary carbohydrate intake does not pose a severe metabolic or nutritional threat, since fat provides sufficient energy and multiple amino acids serve as gluconeogenic substrates (33–35). Via these mechanisms, the body readily shifts between metabolic fuels during carbohydrate restriction to maintain energy and blood glucose (36, 37). At the extreme of this metabolic shift is the ketotic/ketogenic diet, in which carbohydrate restriction prompts a shift to fat-derived ketone bodies as the primary circulating fuel source. Humans can survive and arguably thrive on low-carbohydrate ketogenic diets (38, 39), and available evidence suggests that animals do not adaptively shift towards carbohydrate in response to carbohydrate restriction (40). Thus there is little evidence that carbohydrate intake is necessary per se, or that carbohydrate restriction promotes a specific appetite for carbohydrate that is analogous to either sodium appetite (described above) or protein appetite (described below).

However, 2 separate lines of evidence support a degree of carbohydrate regulation. The first comes from research using the geometric framework, where several studies suggest that animals eat to a carbohydrate target (29, 30, 41). In these tests, the animals are not “defending” carbohydrate intake against restriction in the manner described above, but are instead eating to a carbohydrate target when possible. The second line of evidence comes from studies demonstrating that animals tend to avoid carbohydrates when carbohydrate metabolism is defective or altered, particularly during states of diabetes (42–45). Interestingly, cats appear to show a unique and robust avoidance of excess carbohydrate intake that is consistent with their status as obligate carnivores (46). Collectively, these data suggest that, for most omnivores, if carbohydrate intake is monitored, it is likely at a relatively low priority.

Unlike carbohydrates, there are 2 dietary fatty acids that are considered essential, specifically linoleic acid (omega-6) and alpha-linoleic acid (omega-3), with other species of polyunsaturated fatty acids possibly being conditionally essential. Consistent with this metabolic requirement, at least one publication suggests that restriction of omega-3 fatty acids triggers a preference for omega-3 rich foods (47). There are also anecdotal, historical accounts in which the prolonged consumption of extremely lean meat negatively impacts health and promotes a craving for fat (sometimes termed *rabbit starvation*), suggesting that intake of fat, or perhaps fat-soluble vitamins, is essential. It therefore seems possible that some defense against fat restriction exists, but very little work has been done in this area. Contrastingly, the studies using the geometric framework generally observe either a very weak or nonexistent regulation of fat intake, and as a result, fat acts to dilute protein and carbohydrate and thus increase intake (29, 30, 41). Finally, there is evidence that animals will avoid fat intake in states where fat metabolism is defective/blocked. For instance, mice with a genetic defect in short-chain fatty acid oxidation tend to avoid short-chain fatty acids, while pharmacological blockade of fatty acid oxidation reduces preference for fat (48, 49).

Collectively, the above data provide only limited overall support for a regulatory defense against the specific restriction of carbohydrate or fat intake, with the possible exception of essential fatty acids. Considering the well-accepted and very compelling evidence for an adaptive defense against energy restriction, it seems likely that regulatory systems primarily ensure the consumption of adequate energy while being largely ambivalent as to whether the energy source is carbohydrate or fat. However, this lack of a regulatory defense against fat or carbohydrate restriction does not imply that carbohydrate and fat content do not influence food intake or preferences, quite the contrary. Fat and carbohydrates, particularly sweet foods, are robust drivers of food choice and intake, with humans both preferring and overconsuming diets or foods that are sweet and/or fatty, not due to any specific defense of metabolic need, but instead due to palatability, pleasure, and reward (1, 8, 50, 51).

### Strong behavioral evidence for a defense against protein restriction

In contrast to fat and carbohydrate, there is a large and growing literature that rather clearly demonstrates that animals selectively detect and adaptively respond to the restriction of dietary protein. A large number of recent reviews have discussed this topic, and we will therefore provide a small overview and refer the interested reader to these other excellent resources (32, 52–61).

The manipulation of dietary protein quality and quantity and its impact on food intake and body weight has a long, rich historical background (52). The impact of altered dietary protein has been studied in both laboratory and agricultural models for well over 100 years, and protein intake continues to receive significant emphasis in both the research literature and popular press. Mammals cannot synthesize a subset of amino acids, which are therefore essential in the diet, and it is reasonable to hypothesize that regulatory mechanisms exist that detect dietary protein and/or essential amino acid restriction and adaptively alter metabolism and behavior to compensate for this restriction. Historical work on dietary protein intake and feeding behavior primarily focused on the effects of high-protein diets and amino acid imbalance (essential amino acid restriction). High-protein diets tend to suppress food intake in both humans and rodents (54, 55, 62–65), although some studies suggest that rats will consume and grow rather well on high-protein diets if given sufficient time to adapt to the diet (63, 66–68). A severe amino acid imbalance has also been shown to suppress food intake (65, 69, 70) by producing a learned aversion (71) that can be conditioned by both the diet and cues associated with the diet (72). Although a focus on low-protein diets is historically less frequent, a number of studies demonstrate that low-protein diets induce hyperphagia, particularly in rodent models (73–79). However, it must be noted that this effect is not observed universally (68, 80), that it can vary depending on the age and metabolic needs of the animal (75), and that at extremely low protein content the hyperphagia is abandoned and food intake decreases (68, 81, 82). Importantly, there are no clear definitions for “normal,” “low,” or “extremely low” protein, in part because factors such as strain, sex, age, and metabolic state influence physiological protein need and thus the amount of protein required in the diet.

Although animals tend to increase total food intake when housed on a single, low-protein diet, it is important to note that protein restriction does not necessarily trigger generalized hyperphagia. When given a choice, a wide range of species will self-select between diets that are high and low in protein in order to maintain protein intake (83–92). Protein restriction also induces a specific selection for protein, marked by protein-specific increases in preference and motivation (77, 80, 92–100). Similar observations have been made in humans (101, 102). These data collectively suggest that protein restriction triggers a specific appetite for protein, although it is currently unclear whether this appetite is innate (like sodium appetite) or instead a learned response that is dependent on experiencing the postingestive consequences of the diet (92, 103). The concept of a protein-specific appetite meshes well with multiple studies utilizing nutritional geometry and the geometric framework. These data strongly argue that a number of species defend protein intake, eat to a protein target, and prioritize protein over fat or carbohydrate intake (29, 30, 41, 104). The observation that protein is prioritized and regulated to a specific target suggests that small changes in the proportion of dietary protein can induce large changes in total food and energy intake, a concept termed *protein leveraging* (29, 30, 32, 56, 105–108). Collectively, these data provide a convincing argument, not only that dietary protein content influences feeding behavior, body weight, and metabolism, but more particularly that protein intake is regulated and defended in a manner at least analogous to energy intake. This behavioral evidence therefore prompts the obvious question: What are the cellular, endocrine and/or neural mechanisms that underpin this behavioral response to protein restriction?

## FGF21 as a Regulator of Macronutrient Preference

The fibroblast growth factor (FGF) family is composed of a large number of secreted proteins that influence an array of physiological and cellular functions (109). FGF21 is a member of a small subgroup of FGFs, along with FGF15/19 and FGF23, known as the “endocrine” FGFs that circulate in appreciable amounts within the bloodstream and act as true endocrine hormones (110, 111). Cellular FGF21 signaling is mediated by a receptor complex that includes a classic FGF receptor (FGFR1c) and a co-receptor known as beta-Klotho (Klb), with Klb functioning as the primary binding/targeting factor providing cellular specificity and FGFR functioning as the catalytic subunit that drives intracellular signaling (112, 113). FGF21’s ability to reduce body weight, glucose levels, and lipid concentrations in models of obesity generated substantial initial interest (114–116). Since this early work, FGF21 has been linked to a variety of metabolic states, diseases, and physiological endpoints, and a large number of prior reviews cover this large and at times complex literature (117–125).

### Nutritional regulation of FGF21: increased by high carbohydrate and low protein

Although initially identified as a fasting hormone (126–130), recent work suggests that the nutritional regulation of FGF21 is much more complex and nuanced. The effect of fasting and ketogenic diets to increase FGF21 is not nearly as robust in humans as initially observed in mice (131–133), FGF21 is also increased in settings of obesity (132, 134, 135), and more recent work has led to the suggestion that FGF21 is more appropriately a signal of metabolic or cellular stress (136). From a nutritional standpoint, liver FGF21 production seems to be robustly stimulated by an imbalance in macronutrients, particularly settings of high-carbohydrate but low-protein intake. Both acute carbohydrate ingestion and long-term exposure to high-carbohydrate diets significantly increase liver FGF21 mRNA expression and circulating FGF21 levels (137–145), driven at least in part by the transcription factor carbohydrate response element binding protein (ChREBP), which binds directly to the FGF21 promoter (137, 140, 142, 144). Conversely, work from our lab and others indicates that liver FGF21 expression and circulating FGF21 protein levels are increased by the restriction of protein intake in mice, rats, and humans (82, 141, 145–152), with FGF21 also being increased by the restriction of individual amino acids (148, 153–157). These effects appear to be mediated by a mechanism that is different from carbohydrate ingestion, as the FGF21 promoter contains amino acid response elements (AARE) and appears to be regulated at least in part by the classic integrated stress response pathway (GCN2, PERK, ATF4, etc) during both amino acid restriction and endoplasmic reticulum stress (153, 156, 158–161).

Importantly, the effect of high carbohydrate and low protein to increase FGF21 appears to be regulated independently. From an experimental standpoint, the ability of carbohydrate intake to increase FGF21 occurs independently of protein status or intake (143). Similarly, protein restriction is sufficient to increase FGF21 in both settings of high or low carbohydrate (77, 145, 146, 162, 163). While it is not completely clear whether low protein and high carbohydrate in fact synergize to maximally drive FGF21, work from Solon-Biet and colleagues (145) provides the best test of this question. By measuring FGF21 levels in mice consuming a wide range of diets, their data suggest that protein intake is the primary driver of circulating FGF21 levels, but that maximal FGF21 occurs in mice consuming diets that were both low in protein and high in carbohydrate.

Finally, it is also relevant that another nutritional intervention was shown to robustly increase FGF21: alcohol consumption. Ample evidence, in both rodents and humans, demonstrates that ethanol intake increases FGF21 production, largely by the liver (123, 164–169). Currently the mechanism through which alcohol drives this effect is not fully clear. FGF21 has been directly linked to metabolic stress and liver steatosis (132, 136, 170, 171), and thus it seems possible that metabolic effects of alcohol on the liver could underlie this effect. It is also notable that alcohol is often considered the “fourth macronutrient,” with alcohol intake contributing substantially to total caloric intake in many individuals. Therefore, while the physiological/teleological basis for an increase in FGF21 following alcohol consumption is unclear, it is clearly relevant in terms of both nutritional regulation and metabolic health.

### FGF21 inhibits sweet and alcohol intake

The first studies linking FGF21 to effects on macronutrient preference stem from human genetic linkage experiments. These 2 original studies identified variants in the FGF21 locus that were associated with protein, carbohydrate, and fat intake (172, 173), although the relative strength of the association for each individual macronutrient varied somewhat between studies. Additional studies have since supported this relationship between FGF21 and macronutrient intake, including associations with alcohol intake (166, 174, 175). Similar linkages have also been found in the FGF21 co-receptor Klb, although these associations have primarily focused on alcohol intake (176–178). Thus there appears to be a clear link between FGF21 and Klb variants and human macronutrient consumption.

A functional link between FGF21 and macronutrient preference was first demonstrated when 2 simultaneously published manuscripts demonstrated that FGF21 acts in the brain, via Klb, to suppress sweet and alcohol intake (144, 179). The work by von Holstein-Rathlou and colleagues indicated that FGF21-deficient mice increased their consumption of a solid, high-sucrose diet, as well as solutions containing sucrose, glucose, or fructose. Conversely, mice that genetically overexpressed or were treated with FGF21 reduced their consumption of a high-sucrose diet and reduced their consumption of sucrose and the noncaloric sweeteners saccharin and sucralose. This effect of FGF21 on sweet intake was impaired when the FGF21 co-receptor Klb was deleted from the hypothalamic paraventricular nucleus (PVN). Interestingly, these FGF21-dependent effects appeared to be specific for sweet/sugar, as there was no effect on the nonsweet carbohydrate maltodextrin. Similar observations were made by Talukdar and colleagues, who demonstrated that transgenic FGF21 overexpression reduced sucrose, saccharin, and alcohol intake (179). This effect on sweet intake could be reproduced by exogenous FGF21 administration in control mice, but not mice lacking Klb in the brain. Finally, the inhibition of saccharin was reproduced in monkeys treated with an FGF21 analog.

Importantly, this core effect of FGF21 to suppress sweet and alcohol intake has since been replicated by additional studies (164, 180, 181). Currently the mechanisms underlying this effect remain unclear, although multiple studies implicate the central nervous system as the primary site of action (144, 179, 181). The best evidence currently supports the hypothalamic PVN, as the deletion of Klb from the PVN blocked the effects on sweet intake (144). A recent manuscript demonstrated that PVN oxytocin neurons influence sweet intake, express Klb, and are responsive to FGF21 (181). These data suggest that oxytocin neurons might be the neural mediator of the effect of FGF21 on sweet intake, although it should be noted that the work never directly demonstrated that FGF21-dependent changes in sweet intake require signaling in oxytocin neurons (181). Collectively, these data provide compelling evidence that FGF21 acts directly in the brain to suppress both sweet and alcohol consumption and preference (123, 125, 182).

### FGF21 increases protein intake and is required for adaptive shifts in protein preference

While FGF21 clearly influences the intake of carbohydrates, specifically sweet flavors, a separate literature provides strong evidence that FGF21 is critical for the detection of protein restriction (61). As noted above, a rather large number of studies now demonstrate that reductions in dietary protein intake—or the intake of specific amino acids—increase liver FGF21 mRNA expression and circulating FGF21 protein levels (82, 141, 145–157). This increase in FGF21 is directly related to reduced protein intake, as it occurs in settings of both low and high carbohydrate and low and high fat (77, 145, 146, 162, 163). More importantly, our lab and others have established that this increase in FGF21 is absolutely required for metabolic responses to protein restriction. Although wild-type mice exhibit changes in growth, body composition, food intake, energy expenditure, and glucose metabolism in response to dietary protein restriction, these effects are absent in mice that lack FGF21 (77, 146, 148, 160, 183). Most recently, we recapitulated this phenotype by deleting Klb from the brain, strongly arguing that the metabolic response to protein restriction requires FGF21 acting directly in the brain (77).

This evidence that FGF21 is necessary for animals to sense and/or respond to protein restriction obviously raises the question of whether FGF21 might also influence protein intake. The first evidence to support a specific effect on protein intake stems from work by Larsen and colleagues (184), who tested the impact of FGF21 treatment in mice faced with a series of macronutrient preference tests. In the classic 3-macronutrient choice test (where each macronutrient is offered in pure form), systemic FGF21 treatment significantly increased protein intake and decreased carbohydrate intake, without altering fat or total food intake. Then mice were offered a series of diet pairs in which one macronutrient was fixed but the mice could select between the 2 remaining macronutrients. When either fat or carbohydrate was fixed, FG21 significantly increased the consumption of the protein-rich diet. Contrastingly, when protein was fixed and mice selected between carbohydrate vs fat-rich diets, FGF21 treatment did not alter preference. Importantly, this ability of FGF21 treatment to shift preference toward protein was lost in mice lacking Klb in the brain, again suggesting that FGF21 acts in the brain to produce a protein preference.

Considering our own work suggesting that FGF21 is essential for animals to detect and respond to protein restriction (146, 160, 183), our lab was simultaneously working to determine if FGF21 action in the brain was necessary for the physiological shifts in macronutrient preference that were observed during protein restriction. Our data are consistent with that of Larsen, as we observed that FGF21 administration directly into the brain was sufficient to shift preference away from low-protein and towards high-protein mixed diets (77). We then extended this work by demonstrating that protein restriction induces a unique and selective appetite for protein, but that this adaptive, physiological shift in macronutrient preference was lost in mice lacking either FGF21 or Klb in the brain. Finally, this protein preference could be induced in control mice by direct administration of FGF21 into the brain (77). Available evidence therefore strongly indicates that FGF21 signaling in the brain is not only sufficient to increase protein intake, but that brain FGF21 signaling is absolutely necessary for mice to exhibit physiologically adaptive increases in protein intake in response to protein restriction.

## Unanswered Questions and Future Directions: The Brain and Beyond

The above data collectively highlight a fundamentally new physiological role for the metabolic hormone FGF21, and also identify a new mechanism for the regulation of macronutrient preference. However, as with all new discoveries, there are many unanswered questions. Perhaps the most salient is the site of FGF21 action in the brain. As described above, FGF21 signaling depends on the co-receptor Klb (112, 113), and early work defining the location of Klb expression highlighted the suprachiasmatic nucleus (SCN) and brainstem (185, 186). Subsequent studies using RNAScope technology have confirmed expression in the mouse SCN but not the hindbrain, identified sparse expression in other hypothalamic sites (PVN), identified Klb expression in novel brain sites (reticular thalamus, principal sensory, medial trigeminal neurons, and hippocampal CA1-CA3 transition zone), and suggested some difference in expression between the mouse and nonhuman primate brain (187). Most studies testing central FGF21 action have used intracerebroventricular FGF21 injection or broad, neural-specific Cre drivers (Camk2a-Cre or Synapsin-Cre) to delete Klb from the brain (77, 144, 184–186, 188, 189). While these studies strongly implicate the brain, they provide little insight into the site of FGF21 action. Despite strong Klb expression in the SCN, to date there have no studies that directly tested the functional role of FGF21 signaling exclusively in the SCN. Contrastingly, several studies have implicated the PVN as a site mediating effects on either macronutrient preference or glucose homeostasis (144, 181, 188, 190, 191). Thus, there remains significant uncertainty regarding the site of FGF21 action in the brain, as well as the identity of the neurons and neural circuits that mediate its effects on feeding behavior and metabolism.

Beyond this focus on the brain, additional questions remain regarding the mechanisms through which FGF21 influences both feeding behavior and metabolism. First, the physiological settings and underlying mechanisms through which these various nutritional signals (alcohol, carbohydrate, and protein) interact to regulate FGF21 expression and secretion are unclear. This includes not only signaling events in the liver but also the extent to which, for instance, carbohydrate-dependent increases in FGF21 produce similar downstream responses to those induced by alcohol or low protein. A similar question is the extent to which the regulation of alcohol, sweet, and protein preference is mediated by overlapping or distinct brain areas, as it seems unlikely that these rather diverse behavioral responses might be mediated by the same neural circuits. A third question relates to how FGF21-dependent signaling interacts with classic signals of energy balance (leptin, AgRP neurons, etc). Leptin is often considered a prototypical signal of energy balance, yet a rather large data set suggests that FGF21 is not influenced by energy intake or status. Do these 2 hormones engage overlapping or diverse neural pathways? Do they interact in any synergistic or antagonistic way? Another question is whether FGF21 also plays any role in the adaptive response to high-protein diets, which are well accepted to suppress feeding. There is some discrepancy in the literature, but at least 2 publications suggest that FGF21 is inhibited by high protein intake (151, 192). Finally, it will be important to test the extent to which the effects of FGF21 to influence macronutrient preference and food choice translate to humans.

## Summary

**Figure 1.**
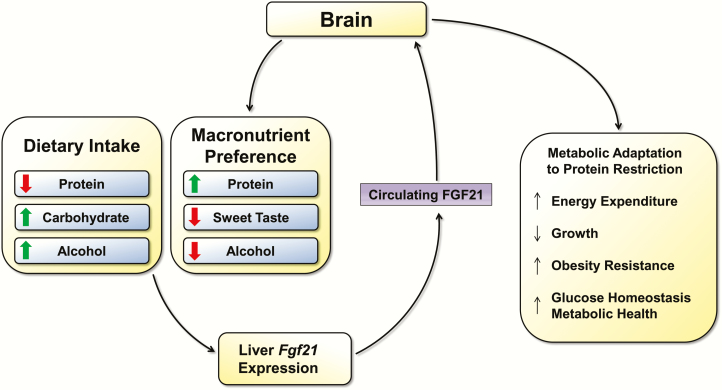
Interaction between dietary macronutrient balance and FGF21. Diets that contain either low levels of protein or high levels of carbohydrate or alcohol trigger increases in liver FGF21 expression and circulating FGF21 protein levels. Although FGF21 may act on multiple tissues, the brain appears to be a primary mediator. Central FGF21 has been shown to directly suppress preference for sweet and alcohol yet increase protein intake and preference. Central FGF21 is also required for adaptive metabolic responses to protein restriction.

The above data provide strong evidence that FGF21 mediates a clear and consistent change in macronutrient preference. FGF21 sequence variants are linked with alterations in macronutrient preference and alcohol intake in humans, while FGF21 directly suppresses sweet and alcohol intake but increases protein intake. These effects on feeding behavior are also highly consistent with the nutritional regulation of FGF21, as FGF21 is increased by high carbohydrate/alcohol intake and by low protein intake. Importantly, these effects of FGF21 are not simply the result of pharmacological treatment, as physiological shifts in macronutrient preference during dietary protein restriction depend on FGF21 and its ability to signal in the brain. Based on this large and growing data set, it seems likely that a primary physiological role for FGF21 is to serve as a signal of macronutrient imbalance (Fig. 1), specifically the excessive consumption of foods that are rich in carbohydrate but poor in protein (relatively common in nature). Such diets do not trigger traditional energy balance signals because no energy restriction is occurring, but instead produce a significant increase in FGF21. FGF21, via its effects in the brain, acts to suppress the consumption of carbohydrate (particularly sweet) while increasing the consumption of protein. Importantly, in this model, FGF21 fulfills a physiological and nutritional role that is unique from other known nutritional hormones, since it does not act to regulate food intake generally but instead to mediate adaptive changes in both metabolism and behavior in response to excess consumption of carbohydrate and alcohol, and/or reductions in the intake of protein intake.

## Abbreviations

ChREBP: carbohydrate response element binding protein
FGF21: fibroblast growth factor 21
FGFR: fibroblast growth factor receptor
Klb: beta-Klotho (co-receptor)
PVN: paraventricular nucleus
SCN: suprachiasmatic nucleus

## Additional Information

***Disclosure Summary:*** The authors have no conflicts to disclose.
